# SETD2 epidermal deficiency promotes cutaneous wound healing via activation of AKT/mTOR Signalling

**DOI:** 10.1111/cpr.13045

**Published:** 2021-05-05

**Authors:** Xiaoxue Li, Changwei Liu, Yiwen Zhu, Hanyu Rao, Min Liu, Liming Gui, Wenxin Feng, Huayuan Tang, Jin Xu, Wei‐Qiang Gao, Li Li

**Affiliations:** ^1^ State Key Laboratory of Oncogenes and Related Genes School of Medicine and School of Biomedical Engineering Renji Med‐X Clinical Stem Cell Research Center Ren Ji Hospital Shanghai Jiao Tong University Shanghai China; ^2^ School of Biomedical Engineering and Med‐X Research Institute Shanghai Jiao Tong University Shanghai China; ^3^ School of Rehabilitation Science Shanghai University of Traditional Chinese Medicine Shanghai China; ^4^ State Key Laboratory of Chemical Oncogenomics School of Chemical Biology and Biotechnology Peking University Shenzhen Graduate School Shenzhen China

**Keywords:** AKT, cutaneous wound healing, histone modification, keratinocytes, mTOR Pathway, SETD2

## Abstract

**Objectives:**

Cutaneous wound healing is one of the major medical problems worldwide. Epigenetic modifiers have been identified as important players in skin development, homeostasis and wound repair. SET domain–containing 2 (SETD2) is the only known histone H3K36 tri‐methylase; however, its role in skin wound healing remains unclear.

**Materials and Methods:**

To elucidate the biological role of SETD2 in wound healing, conditional gene targeting was used to generate epidermis‐specific *Setd2*‐deficient mice. Wound‐healing experiments were performed on the backs of mice, and injured skin tissues were collected and analysed by haematoxylin and eosin (H&E) and immunohistochemical staining. In vitro, CCK8 and scratch wound‐healing assays were performed on *Setd2*‐knockdown and *Setd2*‐overexpression human immortalized keratinocyte cell line (HaCaT). In addition, RNA‐seq and H3K36me3 ChIP‐seq analyses were performed to identify the dysregulated genes modulated by SETD2. Finally, the results were validated in functional rescue experiments using AKT and mTOR inhibitors (MK2206 and rapamycin).

**Results:**

Epidermis‐specific *Setd2*‐deficient mice were successfully established, and SETD2 deficiency resulted in accelerated re‐epithelialization during cutaneous wound healing by promoting keratinocyte proliferation and migration. Furthermore, the loss of SETD2 enhanced the scratch closure and proliferation of keratinocytes in vitro. Mechanistically, the deletion of *Setd2* resulted in the activation of AKT/mTOR signalling pathway, while the pharmacological inhibition of AKT and mTOR with MK2206 and rapamycin, respectively, delayed wound closure.

**Conclusions:**

Our results showed that SETD2 loss promoted cutaneous wound healing *via* the activation of AKT/mTOR signalling.

## Graphical abstract

The epidermis‐specific *Setd2*‐deficient mice showed accelerated re‐epithelialization during cutaneous wound healing by promoting keratinocytes proliferation and migration. Mechanistically, deletion of *Setd2* resulted in the activation the AKT/mTOR signalling pathway and pharmacological inhibitions of AKT and mTOR with MK2206 and rapamycin delayed wound closure, respectively.

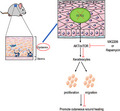

## 1 INTRODUCTION

Mammalian skin is composed of the inner and outer epidermis, separated by a basement membrane.^1^ The skin is a natural physical and immune protective barrier that prevents not only the penetration of harmful microorganisms, but also dehydration.^2^, ^3^ Once the skin barrier is damaged, wound‐healing process begins immediately. Cutaneous wound healing is a complex and dynamic process that involves three phases: inflammation, re‐epithelialization and tissue remodelling.^4^, ^5^ Even though these three phases occur sequentially, they overlap, and the extracellular matrix (ECM), soluble growth factors and multiple cell types, such as immune cells, fibroblasts and keratinocytes (KCs), participate in these phases.^6^ Re‐epithelialization is the formation of new epithelium and covering of the wound surface, which requires the migration and proliferation of KCs.^7^

Epigenetic regulation controls the transcriptional activation or silencing of genes without changing the DNA sequence, governing the phenotypic plasticity of individual cells, organs or a whole organism.^8^ In the skin, epigenetic regulation mechanisms play an important role in its development, homeostasis and wound repair.^9^ Histone modification is a type of epigenetic regulation that affects the transcriptional activity of genes and is involved in normal physiological processes and diseases.^10^, ^11^ Several studies have reported that histone modification and related enzymes play an essential role in skin wound healing. For example, H3K27me3 demethylase JMJD3 interacts with NF‐κB, resulting in increased expression of inflammatory, matrix metalloproteinase and growth factor genes, while the inactivation of JMJD3 leads to delayed wound healing.^1^, ^12^ The hair follicle cells of mice lacking EZH1 and EZH2 histone H3K27 tri‐methylases have defective cell proliferation and wound healing, even though epidermis continues to hyperproliferate.^13^ Furthermore, the hypomethylation of histone H3 K4/9/27me3 is beneficial for the differentiation and growth of hair follicles (HFs) and promotes wound healing.^14^

SETD2 (SET domain‐containing protein 2) was first identified as the protein associated with Huntington's disease (HD).^15^ Currently, SETD2 is the only known H3K36 tri‐methylase; it interacts with RNA polymerase II to mediate transcriptional extension, resulting in changes in gene transcription levels.^16^, ^17^ As a tumour suppressor, SETD2 plays an important role in gene transcription regulation, DNA damage repair and alternative splicing.^18^, ^19^, ^20^, ^21^, ^22^ Recently, SETD2 has been extensively studied in various biological processes and diseases. Loss of SETD2 function has been investigated in several human tumours, including GI stromal tumours, renal cell carcinoma, pancreatic ductal adenocarcinoma (PDAC), prostate cancer, breast cancer, leukaemia and high‐grade gliomas.^23^, ^24^, ^25^, ^26^, ^27^, ^28^, ^29^ Moreover, the role of SETD2 in hematopoietic stem cell self‐renewal, sperm development, bone marrow mesenchymal stem cell differentiation, V(D)J recombination, maternal epigenome and embryonic development has been examined.^30^, ^31^, ^32^, ^33^, ^34^ In addition, four non‐histone substrates of SETD2: α‐tubulin, STAT1, EZH2 and actin were discovered.^23^, ^35^, ^36^, ^37^ However, the role of SETD2, an important histone‐modifying enzyme, in skin wound healing is still not understood.

In this study, to investigate the role of SETD2 in skin wound healing, we generated epidermis‐specific *Setd2*‐knockout mice and showed that SETD2 deficiency promoted cutaneous wound healing through the activation of AKT/mTOR pathway.

## 2 MATERIALS AND METHODS

### 2.1 Mice

*Setd2^fl/fl^* mice were generated by Shanghai Biomodel Organism Co. using conventional homologous recombination in embryonic stem (ES) cells as previously described.^19^, ^29^ Tg‐CK5CreERT2; R26R‐CAG‐lsl‐Tomato mice were purchased from the Jackson Laboratory. *Setd2^‐KO^* mice were generated by crossing *Setd2*‐floxed mice with K5CreERT2 mice, and tamoxifen was intraperitoneally injected at 100 mg kg^−1^ body weight. All mice were maintained in a specific‐pathogen‐free (SPF) facility, and all experimental procedures were approved by the Animal Ethics Committee of School of Biomedical Engineering & Med‐X Research Institute, Shanghai Jiao Tong University. Primers used for genotyping are listed in Table S1.

### 2.2 In vivo wound‐healing experiments

Mice (8 to 10‐week‐old littermates) were anaesthetized by intraperitoneal injection of tribromoethanol, and their backs were shaved. Four 4‐mm full‐thickness cutaneous biopsy punch wounds were made on the back of each mouse. The entry wounds were photographed on days 0, 1, 3, 5, 7 and 9. The wound areas were measured using ImageJ software. Mice were euthanized 1, 3 and 5 days after wounding by carbon dioxide inhalation, the wounds were excised, fixed overnight at 4°C with 4% paraformaldehyde and then embedded in paraffin.

### 2.3 Isolation of primary mouse keratinocytes

Primary mouse keratinocytes were isolated from the skin as previously described.^38^, ^39^ Briefly, the skin of 10‐week‐old mice was treated with dispase II (Gibco,17105‐041) overnight at 4°C. The epidermis was separated and digested for 10 min at 37°C with 0.25% trypsin/EDTA (Gibco, 25200‐072) and strained through a 70‐μm filter. The supernatants were centrifuged, and cells were collected.

### 2.4 Cell culture

Human immortalized HaCaT keratinocyte cell line^40^ was cultured in DMEM supplemented with 10% foetal bovine serum (FBS) and 1% penicillin‐streptomycin at 37°C in a humidified 5% CO_2_ atmosphere.

### 2.5 Histology, H&E staining and immunohistochemistry (IHC)

Tissues were fixed in 4% paraformaldehyde overnight at 4°C, dehydrated and embedded in paraffin. Sections (5 μm) were cut and stained with haematoxylin and eosin (H&E). For IHC staining, sections were deparaffinized, rehydrated, subjected to antigen retrieval in citrate buffer, and endogenous peroxidases were quenched with 3% H_2_O_2_. Blocking was performed with 5% BSA for 1 hour at room temperature. Next, the samples were incubated with primary antibodies for 12‐16 hours at 4°C. The primary antibodies used were anti‐Ki67 (Abcam, ab15580) and anti‐K5 (Abcam, ab52635). After three washes in PBS, the sections were incubated with an HRP‐conjugated secondary antibody for 1 hour at room temperature and then counterstained with haematoxylin. Images were acquired using a Leica microscope, and staining intensities were calculated using ImageJ software. The antibodies used for staining are listed in Table S2.

### 2.6 Immunofluorescence

Skin samples were fixed in 4% paraformaldehyde for 30 minutes at 4°C, transferred to 30% sucrose overnight and then embedded in OCT. Sections (7 μm) were cut, permeabilized with Triton X‐100 and blocked with 5% BSA. Next, the sections were incubated with primary antibodies (anti‐SETD2 (LS‐C332416), anti‐p‐AKT (CST, #4060) and anti‐p‐mTOR (CST, #5536)) at 4°C for 12‐16 hours, followed by the incubation with the secondary antibodies at 37°C for 1 hour. Nuclei were counterstained with DAPI. All antibodies used for immunofluorescence are listed in Table S2.

### 2.7 Scratch wound‐healing assay

HaCaT cells were plated at 1 × 10^5^ cells/well in triplicate in 6‐well plates. Scratch assays were performed using completely confluent HaCaT cells. Scratches were generated using a 200 μL plastic pipette tip. Suspended cells were washed off, and the remaining cells were cultured in the medium without FBS to inhibit cell proliferation. Images were acquired immediately after scratches were generated, as well as 12 hours and 24 hours after scratching. The plates were washed with PBS before imaging to reduce the number of dead cells in the field of view.

### 2.8 RNA isolation and quantitative RT‐PCR

Total RNA was extracted from cultured cells or tissues using an RNA extraction kit (BioTeke) following the manufacturer's protocol. RNA was reverse transcribed using an RT reagent kit (Takara). The cDNA was subsequently subjected to TB Green‐based real‐time PCR analysis. GAPDH was used to normalize the results, and the data were presented as the mean ± SD. The *P*‐value was calculated using Student's *t* test. The primers used for the qPCR analysis are listed in Table S1.

### 2.9 Western blot analysis and antibodies

Cell and tissue samples were lysed in RIPA buffer (Beyotime, P0013B) supplemented with protease and phosphatase inhibitors (MCE). Protein concentrations were measured using the BCA Protein Assay (Thermo Fisher Scientific). Proteins were separated using 6% and 10% SDS‐PAGE gels and then transferred to polyvinylidene fluoride (PVDF) membranes or nitrocellulose membranes (Millipore). The membranes were blocked with 5% skim milk in TBST for 1.5 hours at room temperature and subsequently incubated with primary antibodies overnight at 4°C, followed by incubation with secondary antibodies for 1 hours. The primary antibodies used in this study were as follows: anti‐SETD2 (LS‐C332416), anti‐H3K36me3 (Abcam, ab9050), anti‐H3 (Abcam, ab10799), anti‐AKT (CST, #2920), anti‐mTOR (CST, #2983), anti‐p‐AKT (CST, #4060), anti‐p‐mTOR (CST, #5536) and anti‐GAPDH (CST, #5174). The antibodies used for Western blotting are listed in Table S2.

### 2.10 Plasmids, transfections and lentiviruses

Human *Setd2* cDNA was generated by polymerase chain reaction and cloned into the pCMV6‐Entry vector with an HA‐tag. HaCaT cells were transfected with EZ transfection reagent according to the manufacturer's instructions. Cell lines transiently expressing exogenous *Setd2* were obtained 24‐48 hours after transfection. shRNA sequences for *Setd2* (sh‐*Setd2*) were cloned into the lentiviral vector pLV‐H1‐SGIPZ. Lentiviral packaging plasmids PSPAX2 and pMD2.G were co‐transfected with the sh‐*Setd2* plasmid into 293T cells for virus production. Virus‐infected cells were selected using 1.5 µg/mL puromycin for 2 weeks to generate stable transfections. sh‐*Setd2* sequences were as follows: 5'‐AAGCAAAGAAGTATTCAGAAATAGTGAAGCCACAGA.

TGTATTTCTGAATACTTCTTTGCTT‐3'.

### 2.11 AKT/mTOR inhibition experiments

*Setd2*‐knockdown HaCaT cells were treated with MK2206 (MCE, HY10358) and rapamycin (MCE, HY‐10219) at concentrations of 10 μmol/L and 50 nmol/L, respectively. In vivo inhibition of AKT and mTOR signalling by MK2206 and rapamycin, respectively, was performed as previously described.^41^, ^42^, ^43^ Rapamycin was injected intraperitoneally (i.p.) at 1 mg/kg every other day. MK2206 was administered via oral gavage at 100 mg/kg per day, three times a week. Rapamycin or MK2206 treatments were initiated 7 days before the wounding experiment.

### 2.12 RNA‐seq and analyses

Skin tissue mRNA was obtained from 12‐week‐old *Setd2^‐KO^* and *Setd2^fl/fl^* mice. Differential gene expression was analysed using DESeq2 package. The list of significance was determined by setting a false discovery rate (FDR) threshold at a <0.05, and |log2FC| > 0.585. All differentially expressed genes were subsequently analysed using GO and pathway analyses.

### 2.13 ChIP‐Seq and analyses

Mouse primary keratinocytes (>1 × 10^7^) were crosslinked with 1% formaldehyde for 5 minutes at room temperature and quenched with 0.125 mol/L glycine. The fragmented chromatin fragments were pre‐cleared and then immunoprecipitated with protein A + G magnetic beads coupled to the anti‐H3K36me3 (ab9050) antibody. After reverse crosslinking, ChIP and input DNA fragments were end‐repaired and A‐tailed using the NEBNext End Repair/dA‐Tailing Module (E7442, NEB) followed by adaptor ligation with the NEBNext Ultra Ligation Module (E7445, NEB). The DNA libraries were amplified for 15 cycles and sequenced using Illumina NextSeq 500 with single‐end 1 × 75 as the sequencing mode. Raw reads were filtered to obtain high‐quality clean reads by removing sequencing adapters, short reads (length <50 bp) and low‐quality reads using Cutadapt (v1.9.1) and Trimmomatic23 (v0.35). Next, FastQC was used to ensure high reads quality. The clean reads were mapped to the mouse genome (assembly GRCm38) using the Bowtie2 (v2.2.6) software. Peak detection was performed using the MACS (v2.1.1) peak finding algorithm with .01 set as *P*‐value cut‐off. Annotation of peak sites to gene features was performed using the ChIPseeker R package.

### 2.14 Statistical analysis

All experiments were repeated at least three times. Unless otherwise indicated, data were presented as the mean ± SD and analysed for statistical significance by two‐way ANOVA or Student's *t* test using GraphPad Prism 8.0.2 software. Statistical significance was set at *P* < .05. *P < .05, **P < .01, ***P < .001 and ****P < .0001.

## 3 RESULTS

### 3.1 Generation of epidermis‐specific *Setd2*‐deficient mice

**FIGURE 1:**
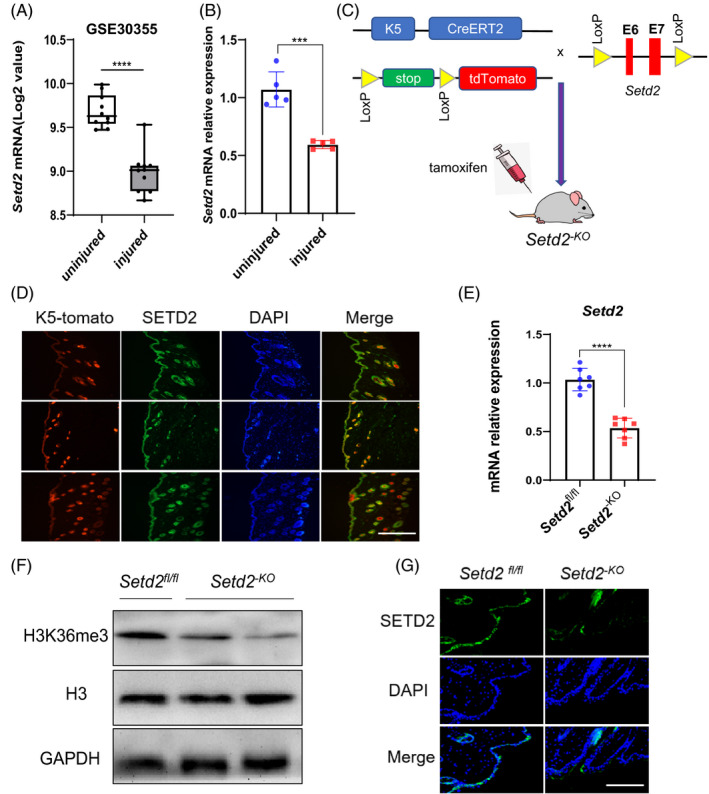
The expression of *Setd2* in KCs and the generation of epidermis‐specific *Setd2*‐deficient mice. A, Box plot of *Setd2* expression levels in normal and injured KCs (using dataset GSE30355; n = 10). B, RT‐qPCR analysis of the *Setd2* mRNA expression in injured and uninjured skin tissues of wild‐type mice (n = 5). C, Schematic representation of generating the *Setd2^‐KO^* mouse model. D, Colocalization of SETD2 (green) and CK5‐Tomato (red) was visualized on frozen skin sections from CK5CreERT2; Tomato adult mice induced by tamoxifen. The nuclei were stained with DAPI (blue). Scale bars: 200 μm. E, RT‐qPCR analysis of *Setd2* mRNA in control and *Setd2^‐KO^* mice (n = 7). F, Western blot analysis of H3K36me3 protein levels. Representative blots are shown. G, Representative immunofluorescence images showing protein expression of SETD2 in *Setd2^‐KO^* and *Setd2^fl/fl^* mice. Scale bars: 200 μm. Data are presented as the mean ± SD; statistical significance was determined using a two‐tailed Student's *t* test, ****P < .0001

To determine whether SETD2 plays a role in re‐epithelialization after injury, we analysed microarray data obtained from the NCBI GEO Datasets GSE30355.^44^ The results showed a decrease in *Setd2* in injured KCs compared to normal KCs (Figure 1A). RT‐qPCR analysis also showed decreased expression of *Setd2* in the full‐thickness wound tissues of wild‐type (WT) mice after injury (Figure 1B). These results suggested that SETD2 played a role in skin wound repair. To further elucidate the role of SETD2 in skin wound healing, we crossed *Setd2*‐flox (*Setd2^fl/fl^*) mice with K5CreERT2 mice to obtain the epidermis‐specific *Setd2* knockout (*Setd2^‐KO^*) mice (Figure 1C).^40^, ^44^ In this system, Cre expression is driven by the K5 promoter, which directs gene expression in the basal cells of the skin, cells believed to be the stem cells of the skin. The immunofluorescence results showed that keratin 5 was mainly expressed in the epidermis and hair follicles; SETD2‐positive cells were also observed in the epidermis and hair follicles (Figure 1D). Furthermore, there was colocalization between SETD2 and keratin 5. Therefore, in our model system, *Setd2* was deleted in K5‐positive cells. We confirmed that the expression levels of SETD2 and H3K36me3 were significantly decreased in *Setd2^‐KO^* mice by RT‐qPCR, Western blotting and immunofluorescent (IF) staining (Figure 1E‐G). *Setd2^‐KO^* mice had no visible skin abnormalities during the growth process until 10 months of age compared to control (*Setd2^fl/fl^*) mice (data not shown).

### 3.2 *Setd2* deficiency promotes cutaneous wound healing and thickens the wound epithelium in mice

**FIGURE 2:**
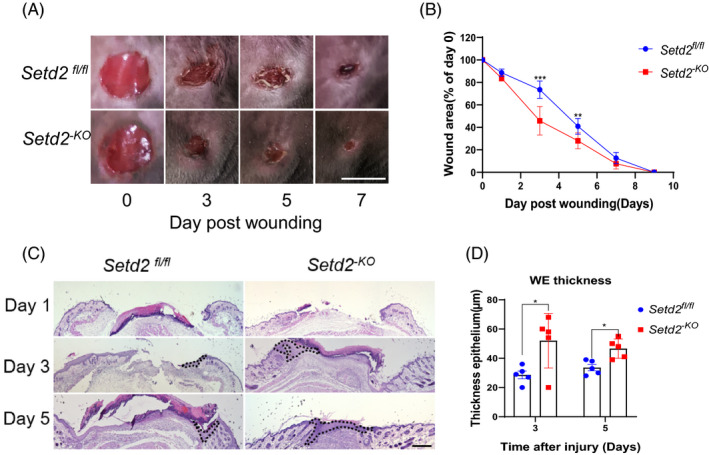
Wound closure is accelerated in the skin of *Setd2^‐KO^* mice. A, Appearance of wound areas of *Setd2^‐KO^* and *Setd2^fl/fl^* mice on days 0, 3, 5 and 7 post‐wounding. Scale bars: 4 mm. B, Quantification of the wound areas. Data are presented as percent wound area at each time point relative to the original wound area in *Setd2^‐KO^* and *Setd2^fl/fl^* mice, n = 8 wounds/group. The analysis was performed using ImageJ software. C, H&E staining of wounds in *Setd2^fl/fl^* and *Setd2^‐KO^* mice at the specified time points after injury. The wound epithelium is depicted by a dotted line. Scale bars: 500 μm. D, Thickness of the wound epidermis in *Setd2^fl/fl^* and *Setd2^‐KO^* mice, n = 5 wounds/groups. Quantification of the thickness was performed using ImageJ software. Data are presented as the mean ± SD; statistical significance was determined using a two‐tailed Student's *t* test, *P < .05, **P < .01 and ***P < .001

Next, we investigated the effect of SETD2 deletion on cutaneous wound healing. Skin punch biopsies were obtained from *Setd2^‐KO^* and age‐matched *Setd2^fl/fl^* (control) mice to assess the wound‐healing process every 2 days for up to 9 days. Three days after wounding, the wound areas in *Setd2^‐KO^* mice were significantly reduced compared to *Setd2^fl/fl^* mice as measured by image analysis, suggesting accelerated wound healing in *Setd2^‐KO^* mice (Figure 2A). Furthermore, the differences in the wound area between *Setd2^‐KO^* and *Setd2^fl/fl^* mice were statistically significant on days 3 and 5 after wounding (Figure 2B). Increased thickness of the wound epithelium (WE) contributed to the re‐epithelialization process. The H&E staining of injured tissues showed that the thickness of WE was significantly increased in *Setd2^‐KO^* mice compared to control mice (Figure 2C,D). These results indicate that the absence of *Setd2* accelerates skin wound healing in mice.

### 3.3 **Accelerated wound closure in** *Setd2^‐KO^* **mice is associated with enhanced keratinocytes proliferation and migration**

**FIGURE 3:**
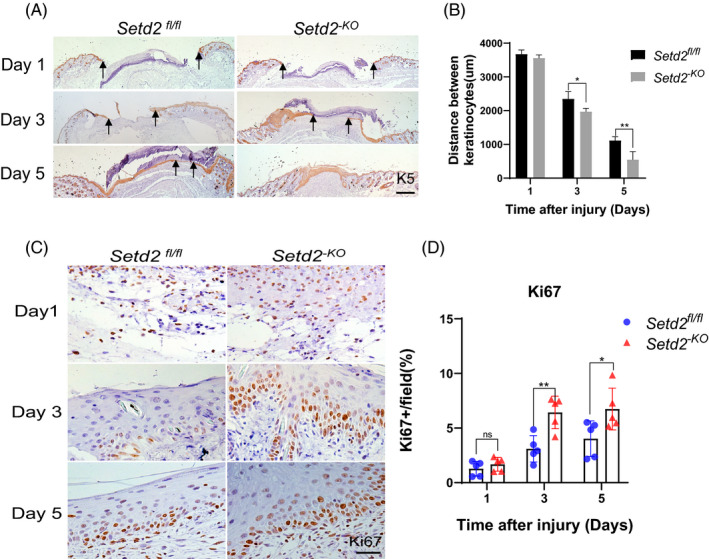
*Setd2* deficiency leads to accelerated KC proliferation and migration during wound healing A, K5 staining (brown) of wounds in *Setd2^fl/fl^* and *Setd2^‐KO^* mice at the specified time points after injury. Arrows indicate the edges of migrated KCs. Scale bars: 500 μm. B, Quantification of the distance between the KCs in the wounds of *Setd2^fl/fl^* and *Setd2^‐KO^* mice; n = 5 wounds/group. C, Immunohistochemical staining using anti‐Ki67 antibody performed on *Setd2^fl/fl^* and *Setd2^‐KO^* wound sections collected on days 1, 3 and 5. Representative images. Scale bars: 50 μm. D, Quantification of Ki67‐positive KCs; data are presented as the percentage of total KC number; n = 5 wounds/group. Data are presented as the mean ± SD; and statistical significance was determined using a two‐tailed Student's *t* test; n.s, no significance, *P < .05, **P < .01 and ***P < .001

Since KC proliferation and migration are involved in the re‐epithelialization process, the process essential for wound healing, ^45^, ^46^, ^47^ and SETD2 is primarily expressed in skin KCs, we further hypothesized that accelerated wound healing is caused by the increased proliferation and migration of these cells. To test this hypothesis, we evaluated the differences in the migration rate of KCs from *Setd2^‐KO^* and *Setd2^fl/fl^* mice by K5 immunostaining. KCs migrated from the edge to the centre of the wound in both *Setd2^fl/fl^* and *Setd2^‐KO^* mice. However, re‐epithelialization occurred significantly faster in *Setd2^‐KO^* mice compared to control mice (Figure 3A). As shown in Figure 3B, the distance between the migration edge in *Setd2^‐KO^* KCs was significantly shorter than in controls. Next, to determine whether accelerated wound closure was due to increased cell proliferation, we used Ki67 (a marker of proliferation) immunohistochemical staining and counted the number of Ki67‐positive KCs in wound areas on different days. The results demonstrated that cell proliferation was significantly enhanced on days 3 and 5 after injury (Figure 3C,D). These results confirmed that *Setd2* deletion accelerated the proliferation and migration of KCs in vivo.

### 3.4 Loss of *Setd2* promotes scratch closure and proliferation of KCs in vitro

**FIGURE 4:**
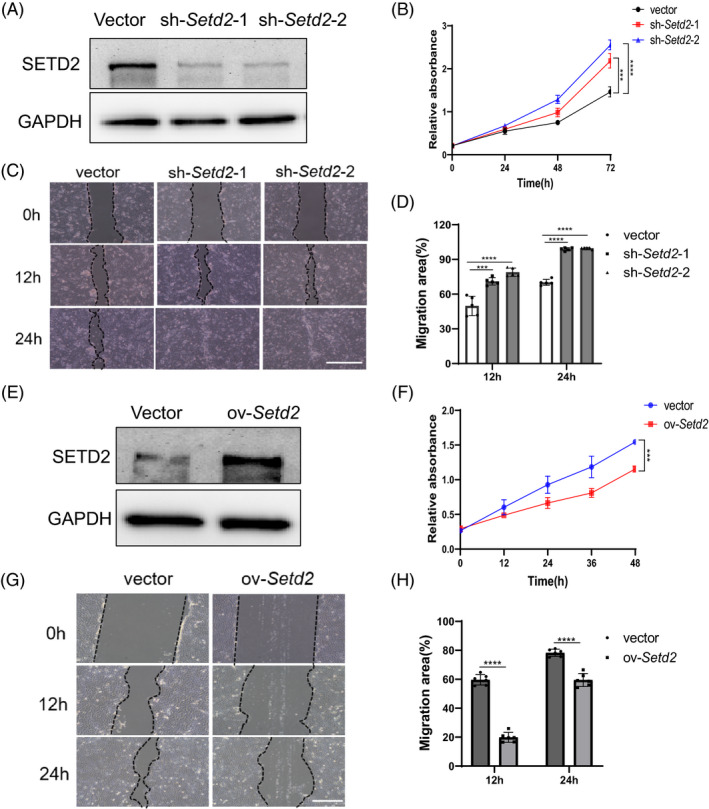
*Setd2* expression levels affect the proliferation and migration of HaCaT cells in vitro. A, Western blot analysis of SETD2 expression in the *Setd2*‐knockdown HaCaT cells transfected either with empty vector or sh‐*Setd2* plasmids. Representative blots are shown. B, CCK8 assay of HaCaT cells transfected either with empty vector or sh‐*Setd2* plasmids. C, Scratch assay of HaCaT cells transfected either with empty vector or sh‐*Setd2* plasmids. The gap closure was significantly accelerated at 12 h and completely closed at 24 h in *Setd2*‐knockdown KCs. Scale bars: 200 μm. D, Quantification of migration area at 12 h and 24 h; n = 5/group. E, Western blot analysis of SETD2 expression in the *Setd2*‐overexpressing HaCaT cells. Representative blots are shown. F, CCK8 assay of HaCaT cells transfected either with empty vector or HA‐*Setd2* plasmids. G, Cell migration ability of *Setd2*‐overexpressing cells compared to the control cells. Scale bars: 100 μm. H, Quantification of migration area; n = 6/group. Data are presented as the mean ± SD; statistical significance was determined using a two‐tailed Student's *t* test and two‐way ANOVA; n.s, no significance, ***P < .001 and ****P < .0001.

To further analyse the effects of SETD2 loss on KC proliferation and migration in vitro, we performed CCK8 and scratch wound‐healing assays on control and *Setd2*‐knockdown HaCaT cells. First, we confirmed that the expression level of SETD2 was significantly decreased after transfecting HaCaT cells with the sh‐*Setd2* plasmid (Figure 4A). As shown by CCK8 assays, *Setd2*‐knockdown promoted cell proliferation (Figure 4B). Scratch assays demonstrated that *Setd2*‐knockdown in HaCaT cells resulted in increased cell migration (Figure 4C,D). Closure of the wound gaps was almost completed by 12 hours and fully achieved by 24 hours in *Setd2*‐knockdown HaCaT cells. This process was significantly delayed in control cells. As expected, *Setd2* overexpression inhibited the proliferation and migration of HaCaT cells (Figure 4E‐H). Therefore, our results further confirmed that *Setd2*‐knockdown accelerated KC proliferation and migration in vitro.

### 3.5 *Setd2*‐deficient skin displays hyperactive AKT/mTOR Signalling

**FIGURE 5:**
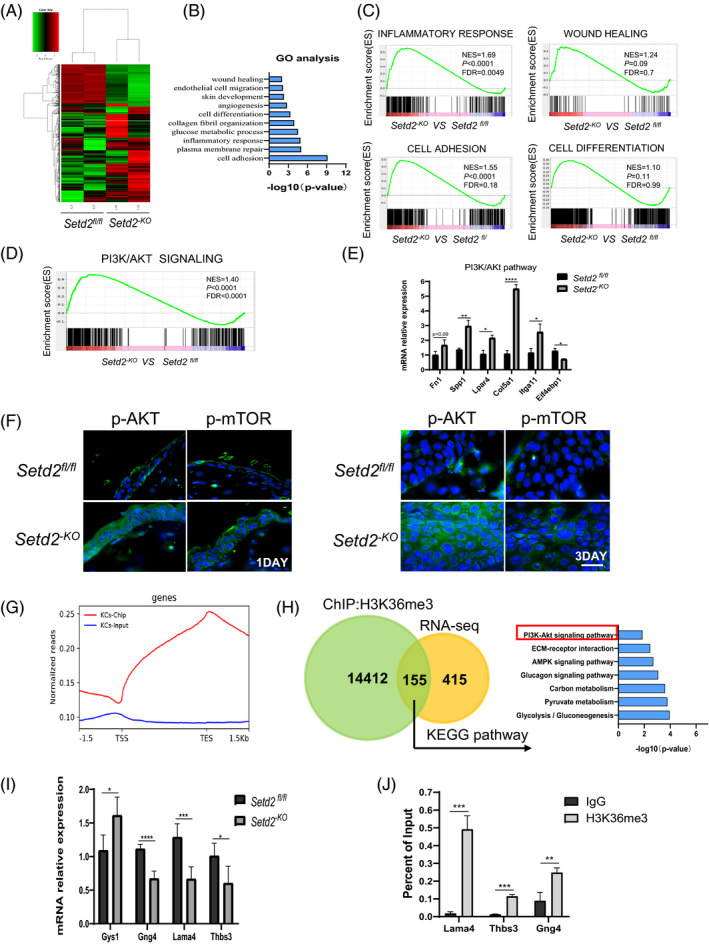
*Setd2*‐deficient skin displays hyperactive AKT/mTOR Signalling A, Heat map of differentially expressed genes in age‐matched *Setd2^fl/fl^* and *Setd2^‐KO^* mice. B, GO term analysis demonstrating upregulated biological processes in age‐matched *Setd2^fl/fl^* and *Setd2^‐KO^* mice. C, GSEA enrichment plots of differentially expressed genes associated with SETD2 deletion. D, GSEA enrichment plots of differentially expressed genes that belong to PI3K/AKT signalling pathway and associated with SETD2 deletion. E, RT‐qPCR analysis of genes related to PI3K/AKT/mTOR signalling. F, Immunofluorescent staining of *Setd2^fl/fl^* and *Setd2^‐KO^* wound sections using anti‐p‐AKT and anti‐p‐mTOR (green) antibodies on days 1 and 3 post‐wounding. Representative images are showing the wound epithelium areas. Nuclei are stained with DAPI (blue). Scale bars: 50 μm. G, Normalized read density of H3K36me3 ChIP‐seq and input signals of primary KCs from *Setd2^fl/fl^* mice, from 1.5 kb upstream of the TSS to 1.5 kb downstream of the TES. H, Venn diagram illustrating H3K36me3 peaks in *Setd2^fl/fl^* primary KCs and the overlap with total differential expression genes determined by RNA sequencing. Right panel shows the KEGG analysis of the overlapping genes. I, RT‐qPCR analysis of PI3K/AKT related gene expression in *Setd2^fl/fl^* and *Setd2^‐KO^* mice as indicated. J, ChIP‐qPCR analysis of H3K36me3 binding for *Gng4*, *Lama4* and *Thbs3* loci in primary KCs from *Setd2^fl/fl^* mice; IgG was used as the control. Data are presented as the mean ± SD; statistical significance was determined using a two‐tailed Student's *t* test; *P < .05, **P < .01, ***P < .001 and ****P < .0001

To understand how SETD2 loss enhances wound healing, we carried out a transcriptomic analysis using skin tissues derived from *Setd2^‐KO^* and control mice at the unwound (day 0) time point to identify early driving events. Clustering analysis showed that the global transcriptome changed dramatically in *Setd2^‐KO^* mice compared to control mice (Figure 5A). Gene ontology (GO) term analysis indicated that SETD2 loss significantly enriched the genes associated with wound healing, including cell differentiation, cell adhesion, inflammatory response, plasma membrane repair and angiogenesis (Figure 5B,C). The GO data were consistent with our phenotypic observations. To elucidate the SETD2‐mediated signalling pathway, we performed gene set enrichment analysis (GSEA). Our results showed that genes related to the PI3K/AKT pathway were significantly enriched in *Setd2^‐KO^* mice (Figure 5D). The mRNA expression levels of these genes were validated using RT‐qPCR (Figure 5E). Furthermore, IF staining showed that the protein expression levels of p‐AKT and p‐mTOR were enhanced in WE areas on days 1 and 3 after injury in *Setd2^‐KO^* mice (Figure 5F). These data suggested that SETD2 deficiency resulted in the activation of AKT/mTOR signalling.

Since SETD2 mainly regulates the expression of downstream genes through H3K36me3, we performed chromatin immunoprecipitation experiments followed by next‐generation sequencing (ChIP‐seq) assays using a H3K36me3‐specific antibody in primary KCs isolated from *Setd2^fl/fl^* mice. H3K36me3 peaks were enriched around the transcription area (Figure 5G). A total of 45 584 H3K36me3 peaks (on 14 567 genes) were identified. To correlate chromatin binding with transcriptional regulation, we integrated ChIP‐seq data with RNA‐Seq data. The results indicated that 155 misregulated genes showed direct H3K36me3 occupancy. Kyoto Encyclopedia of Genes and Genomes (KEGG) analysis revealed that these overlapping 155 genes were functionally associated with ECM‐receptor interaction, AMPK signalling pathway and glucagon signalling pathway, among others. Furthermore, these genes were still closely related to the PI3K/AKT signalling pathway (Figure 5H). RT‐qPCR analysis demonstrated that genes related to the PI3K/AKT signalling pathway, including *Gng4*, *Thbs3* and *Lama4*, were downregulated in *Setd2^‐KO^* compared to *Setd2^fl/fl^* mice (Figure 5I). We further validated the existence of H3K36me3 binding at the gene bodies of *Gng4*, *Lama4* and *Thbs3* by ChIP‐qPCR assay (Figure 5J), and found that the intensity of H3K36me3 binding within these gene loci significantly increased in the H3K36me3 group compared to the IgG group. These results suggested that SETD2 deletion might activate the AKT/mTOR signalling pathway via the regulation of these H3K36me3‐directly occupied genes.

### 3.6 Activation of AKT/mTOR in the epithelial compartment accelerates wound healing

**FIGURE 6:**
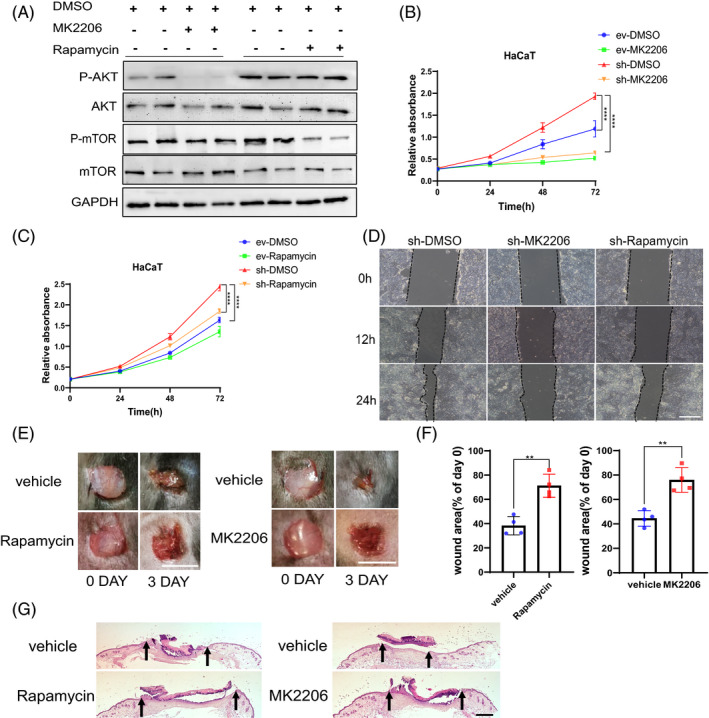
Inhibition of AKT/mTOR signalling pathway suppresses cell proliferation and migration in *Setd2*‐deficient HaCaT cells. A, Western blot analysis of p‐AKT and p‐mTOR expression in *Setd2*‐deficient HaCaT cells treated with either AKT inhibitor MK2206 or mTOR inhibitor rapamycin. B, CCK8 assay of HaCaT cells transfected either with empty vector (ev) or sh‐*Setd2* plasmids (sh) and treated with the AKT inhibitor MK‐2206 or DMSO (vehicle control). C, CCK8 assay of HaCaT cells transfected either with empty vector (ev) or sh‐*Setd2* plasmids (sh) and treated with the mTOR inhibitor rapamycin or DMSO (vehicle control). D, Confluent *Setd2*‐knockdown HaCaT cells were wounded by scraping and treated with MK2206, rapamycin or DMSO (vehicle control). Cell migration towards the wound surface was evaluated at 12 h and 24 h. Scale bars: 100 μm. E, Appearance of wound areas in *Setd2^‐KO^* mice treated either with the vehicle control or inhibitors (rapamycin or MK2206) on days 0 and 3 post‐wounding. Scale bars: 4 mm. F, Quantification of the wound areas. Results are presented as percent wound area at day 3 relative to the original wound area in *Setd2^‐KO^* mice treated with the vehicle control or inhibitors (rapamycin or MK2206); n = 4 wounds/group. The analysis was performed using ImageJ software. G, H&E staining of wound tissues of *Setd2^‐KO^* mice treated either with the vehicle control or inhibitors (rapamycin and MK2206) on day 3 after injury. The arrows indicate the boundary of the wound. Scale bars: 500 μm. Data are presented as the mean ± SD; statistical significance was determined using a two‐tailed Student's *t* test and two‐way ANOVA; **P < .01 and ****P < .0001

To further elucidate the causal link between SETD2 and the AKT/mTOR pathway in wound healing, we transfected HaCaT cells with sh‐*Setd2* plasmids and then treated these cells with an AKT inhibitor (MK‐2206) or an mTOR inhibitor (rapamycin). Western blotting confirmed that the protein levels of p‐AKT and p‐mTOR were decreased in *Setd2*‐knockdown HaCaT cells treated with MK2206 or rapamycin (Figure 6A). As shown by CCK8 assays, *Setd2*‐knockdown promoted cell proliferation, while the treatment with either MK‐2206 or rapamycin significantly inhibited cell proliferation. (Figure 6B,C). Next, scratch assays showed delayed wound closure after the treatment either with MK2206 or rapamycin (Figure 6D). Importantly, *Setd2^‐KO^* mice treated with rapamycin or MK2206 also demonstrated delayed wound closure compared to the vehicle‐treated group. These results indicated that the inhibition of AKT or mTOR in vivo abrogated the accelerated wound healing caused by SETD2 deletion in the epithelial compartment (Figure 6E‐G). Collectively, these results suggest that the accelerated wound healing due to SETD2 deletion is mediated by the activation of AKT/mTOR signalling.

## 4 DISCUSSION

This study investigated the mechanistic role of SETD2 in epidermal KCs during cutaneous wound healing. We identified two novel findings: (a) the deletion of *Setd2* accelerated re‐epithelialization during wound healing *via* increased KC proliferation and migration; and (b) SETD2 deficiency activated AKT/mTOR signalling. Furthermore, the inhibition of AKT/mTOR signalling with MK2206 or rapamycin, significantly reduced KC proliferation and migration. Therefore, we showed, for the first time, that SETD2 plays an essential role in the re‐epithelialization process during cutaneous wound healing.

Histone modification is important for cutaneous wound healing; however, little is known about the role of SETD2, a key histone modifier, in skin wound healing. In this study, we showed that mice lacking *Setd2* had accelerated wound healing. Skin wound healing is a physiological (or a pathological) process in which multiple cells and signalling molecules work together. Previous studies have shown that several signalling pathways, including Wnt, Notch, TGFβ and PI3K/AKT/mTOR, are involved in skin wound healing.^38^, ^48^, ^49^, ^50^ The PI3K/AKT/mTOR signalling pathway plays an important role in numerous diseases, including cancer. This pathway controls several key cellular processes responsible for cellular homeostasis, while aberrant activation of the pathway has been observed in human cancers.^51^, ^52^ In the skin, this pathway is crucial for skin development and homeostasis.^53^ It has been well established that activation of the PI3K/AKT/mTOR pathway can promote cell migration and proliferation, and enhance cutaneous wound healing.^49^, ^54^, ^55^ Therefore, transient pharmacologic activation of the PI3K/AKT/mTOR signalling axis may represent a novel clinical intervention strategy to accelerate the healing of debilitating and life‐threatening wounds.^50^ In our study, we demonstrated that the loss of *Setd2* led to the activation of the AKT/mTOR signalling pathway during wound healing.

RNA‐seq and ChIP‐seq data indicated that seven misregulated genes associated with the PI3K/AKT/mTOR pathway showed direct H3K36me3 occupancies. We verified the mRNA expression levels of these genes including *Fn1*, *Gys1*, *Itga7*, *Pik3r3*, *Gng4*, *Lama4* and *Thbs3* by RT‐qPCR. ChIP‐qPCR validated the existence of H3K36me3 binding at these genes. These results suggested that SETD2 might regulate these downstream genes through H3K36me3, leading to the activation of the AKT/mTOR pathway, and the specific regulatory mechanism needs to be further elucidated.

As a tumour suppressor, SETD2 has been extensively studied in cancers of different tissues. Here, we utilized an animal model of a conditional epidermis‐specific knockout of *Setd2*. However, *Setd2^‐KO^* mice showed no tumour‐associated phenotype in their skin for the first 10 months. Therefore, the biological function of SETD2 in skin cancer could be further studied either by crossing *Setd2^‐KO^* mice with other mutant mice, or by stimulating *Setd2^‐KO^* mice with different environmental factors. In summary, our study contributes to a better understanding of the wound‐healing process and suggests that SETD2 may be considered as a therapeutic target to improve skin wound healing.

## CONFLICT OF INTEREST

The authors declare no conflict of interest.

## AUTHOR CONTRIBUTIONS

XL mainly performed the experiments, analysed the data and wrote the paper. CL and YZ contributed to the animal breeding and identification. HR, ML, LG, WF, HT and JX helped with the experiments. LL and WQG conceived the concept. LL designed the experiments, drafted and revised manuscript. All authors edited and approved the final manuscript.

## Supporting information

Table S1‐S2

## Data Availability

All data are available from the authors upon request. RNA‐Seq and ChIP‐Seq raw data were deposited in the Gene Expression Omnibus (GEO) under accession number GEO: GSE163861.
