# Supplementary material for: SETD2 epidermal deficiency promotes cutaneous wound healing via activation of AKT/mTOR Signalling
Source: Cell Prolif. 2021 May 5;54(6):e13045. doi: 10.1111/cpr.13045 (PMC8168411; doi:10.1111/cpr.13045)
Supplement: Supplementary file 1 — Table S1‐S2 [file CPR-54-e13045-s001.docx]

**Supplementary Table 1: Primers for RT-qPCR, ChIP-qPCR analysis and genotyping.**

**Primers for RT-qPCR**

| **Gene name** | **Sense 5’-3’** | **Antisense 5’-3’** |
| --- | --- | --- |
| mGapdh | AGGTCGGTGTGAACGGATTTG | TGTAGACCATGTAGTTGAGGTCA |
| mSetd2 | CATAGCTGTGAACCAAACTGTGA | TAATTCTGAGCCTGAAGGAACTA |
| mFn1 | ATGTGGACCCCTCCTGATAGT | GCCCAGTGATTTCAGCAAAGG |
| mSpp1 | AGCAAGAAACTCTTCCAAGCAA | GTGAGATTCGTCAGATTCATCCG |
| mLpar4 | AGTGCCTCCCTGTTTGTCTTC | GCCAGTGGCGATTAAAGTTGTAA |
| mCol5a1 | CTTCGCCGCTACTCCTGTTC | CCCTGAGGGCAAATTGTGAAAA |
| mItga11 | TGCCCCAATGGAAACCAATG | CACTCGTGCGACCAGAGAG |
| mEif4ebp1 | GGGGACTACAGCACCACTC | GTTCCGACACTCCATCAGAAAT |
| mGys1 | GAACGCAGTGCTTTTCGAGG | CCAGATAGTAGTTGTCACCCCAT |
| mGng4 | GGCATGTCTAATAACAGCACCA | CACTGGGATGATGAGGGGG |
| mLama4 | ATGAGCTGCAAGGAAAACTATCC | CTGTTTCGTTGGCTTCACTGA |
| mThbs3 | ATGGAGAAGCCGGAACTTTGG | AGTGAGTAAAGCTGTCCGAATCT |

**Primers for ChIP-qPCR**

| **Gene name** | **Sense 5’-3’** | **Antisense 5’-3’** |
| --- | --- | --- |
| Lama4 | GTCTGCCCCCATTACAGAT | AAACAAGAGAGTGCTCGA |
| Thbs3 | ACAGGCAAAACACCCATACAG | CCAAAAAAGGCTTCAGGACC |
| Gng4 | TCTCGCGTCCCCCAAAGGCC | AAAACCTCCCAGTAGGCCC |

**Primers for genotyping**

| **Gene name** | **Sense 5’-3’** | **Antisense 5’-3’** |
| --- | --- | --- |
| K5-Cre | TGATGGACATGTTCAGGGATCGCCA | CCGCCGCATAACCAGTGAAACA |
| Setd2 | GTAAAGTAGTATTATGCCAAGGCCC | TATTTAAACTCTCTCTGGGGGTGG |
| Tomato-red | AAGGGAGCTGCAGTGGAGTA | CCGAAAATCTGTGGGAAGTC |
| Tomato-red | GGCATTAAAGCAGCGTATCC | CTGTTCCTGTACGGCATGG |

Supplementary Table 2: Antibodies

**Antibodies used for IHC(IF) and western blotting**

| **ID** | **Clone/Catalog NO.** | **Dilution** | **Brand** |
| --- | --- | --- | --- |
| SETD2 | C332416 | 1:1000 | LSBio |
| H3K36me3 | ab9050 | 1:1000 | Abcam |
| H3 | ab10799 | 1:1000 | Abcam |
| Ki67 | ab15580 | 1:2000 | Abcam |
| Keratin 5 | ab52635 | 1:500 | Abcam |
| p-AKT | #4060 | 1:1000 | Cell Signaling Technology |
| P-mTOR | #5536 | 1:1000 | Cell Signaling Technology |
| AKT | #2920 | 1:1000 | Cell Signaling Technology |
| mTOR | #2983 | 1:1000 | Cell Signaling Technology |
| GAPDH | #5174 | 1:1000 | Cell Signaling Technology |
